# Editorial: Psychological dimensions of running and other endurance sports, among unexplored socio-cultural groups

**DOI:** 10.3389/fpsyg.2023.1118144

**Published:** 2023-01-24

**Authors:** Patxi León-Guereño, Ewa Malchrowicz-Mośko, Christopher Hautbois, Alfonso Valero-Valenzuela

**Affiliations:** ^1^Department of Physical Activity and Sport Science, Faculty of Education and Sport, University of Deusto, Bilbao, Spain; ^2^Institute of Sport Sciences, Eugeniusz Piasecki Academy of Physical Education, Poznań, Poland; ^3^Faculty of Sport Sciences, Paris-Saclay University, Gif-sur-Yvette, France; ^4^Salud, Actividad Física y Educación Research Group, Sport Sciences Faculty, University of Murcia, Murcia, Spain

**Keywords:** amateur sport, unexplored fields, psychological variables, endurance sports, mass participant sport events

Endurance sports have increased in popularity in recent years, and it is very common to find different mass-participation sporting events around the world attracting thousands of people. While research related to the performance of endurance athletes has been widely addressed with a focus on elite athletes and physiological aspects, studies related to recreational endurance athletes and psychological dimensions should also be properly analyzed. Moreover, despite in recent years researchers have focused on the characteristics of adult athletes, very little has been done in relation to young athletes, the elderly, disabled athletes, or contextual factors such as family or environmental variables, through which we could better understand many of the personal and behavioral characteristics of these athletes.

Many studies have shown that endurance sports are beneficial and have many health benefits for participants. Apart from physical and physiological advantages, the motivations and other psychological variables of endurance athletes, such as anxiety, resilience, mental toughness, or certain personality traits, are increasingly associated with public health. However, apart from adult recreational athletes, the different psychological variables of, for example, older athletes, younger athletes, athletes with different levels of disability and dependence, and people who have suffered from a serious illness such as cancer or COVID-19, have not been analyzed to any great extent. Therefore, it could be said that the characteristics of these different sociocultural groups have not been properly addressed. In addition to these groups, we can also find a gap in the literature in relation to the environmental and contextual factors of the athlete, so the effects of these aspects remain underexplored. Therefore, the aim of the present Research Topic is to collect manuscripts that can be grouped in the analysis of the characteristics of different unexplored sociocultural groups, such as, among others, disabled athletes, amateurs, pregnant women, and people who participate after going through a serious illness such as COVID-19 or cancer, as well as analyzing the influence of cultural variables. Thus, this Research Project aims to fill a gap in the literature.

This special issue includes manuscripts especially related to amateur running, the environmental aspects of this group, the effect of different distances in amateur races, the psychological characteristics of amateur runners (such as wellbeing and anxiety), and customer loyalty in recreational racing. In addition, the motivations and barriers to horseback riding, a practice little-analyzed in literature, have been explored.

Among the studies on running, Thuany et al. examined the effects of individual and environmental characteristics of female runners of different age ranges in Brazil on their performance details. They found that in “adult” runners, only individual characteristics were statistically associated with performance, while environmental characteristics were not significant. For the “older adults” group, both individual and environmental characteristics were associated with their running performance. Based on these findings, the authors suggest that this information could be used in order to guide the development of strategies and projects aimed at increasing the participation of women in running and their involvement in physical activities, through promoting a more friendly environment for them supported by public policies. In another study related to recreational long-distance races, Cabello-Manrique et al. examined runners' loyalty based on their experiences, and the differences between novice and experienced runners were analyzed in relation to quality, value, satisfaction, and loyalty, based on the runners' experiences. The authors found that quality was a direct antecedent of value and satisfaction, while at the same time, value was associated directly with satisfaction and indirectly with loyalty. These results showed that runners' loyalty to a race depends primarily on their satisfaction and the runner's experience.

Gerasimuk et al. examined amateur runners' motivations to practice, depending on their age and running distances. Among these athletes, the motivations of marathoners, ultramarathoners, and non-starters were examined. The results showed that age was associated with athletes' motives to run, and statistical differences were found among marathoners, ultramarathoners, and non-starters. The authors reported that the motives of young non-starters were associated with personal goal achievement, and for both marathon and ultramarathon runners, recognition, and competition were important. Goal achievement was the least important motivation for all groups in older people, while among the oldest runners, the most important motives were self-esteem for non-starters and health orientation for marathoners and ultramarathoners. In another study, Jaenes et al. aimed to explore the various psychological aspects that influence athletic performance in amateur male marathoners, including variables such as anxiety and general wellbeing. The results showed that marathon running improves athletes' wellbeing in four out of five wellbeing dimensions and reduces somatic anxiety and concentration-impairing anxiety. This information can be used in order to design interventions for recreational athletes.

Finally, Sáez et al. contributed to this special issue by examining horseback riding, a practice little-analyzed in the literature. The main objective of this research was to describe horseback riders' health benefits and their barriers to participation in terms of age and their skill level. Results showed that the perceived benefits were significantly different according to the age of the participants, while the differences between amateur and professional riders were significantly connected to barriers in participation. These findings can contribute to a better understanding of equestrianism in order to promote this physical activity as an opportunity to meet international organizations' considerations for active people, and thereby athletes' health.

In conclusion, these studies have focused mainly on adults and older people and the benefits of practicing running, with the exception of one study related to horseback riding. In this sense, a runner's loyalty to a race depends primarily on their satisfaction. Moreover, the motivation to practice running for younger participants can depend on whether individuals are non-starters, marathon or ultramarathon runners. For older runners, non-starters' motives are related to self-esteem, while health orientation is related to marathoners. What is clear is that marathon running improves wellbeing and reduces anxiety. Furthermore, adult female runners' performances are associated with individual characteristics, while older adult females also take environmental aspects into consideration.

## Author contributions

All authors contributed directly to the revision and reading of the work and approved the submitted version for publication.

